# Supplementary material for: Structural basis of lipid-droplet localization of 17-beta-hydroxysteroid dehydrogenase 13
Source: Nat Commun. 2023 Aug 24;14:5158. doi: 10.1038/s41467-023-40766-0 (PMC10449848; doi:10.1038/s41467-023-40766-0)

Structural basis of lipid-droplet localization of 17-beta-hydroxysteroid dehydrogenase 13

Shenping Liu, Ruth F. Sommese, Nicole L. Nedoma, Lucy Mae Stevens, Jason K. Dutra, Liying Zhang, David J. Edmonds, Yang Wang, Michelle Garnsey, Michelle F. Clasquin

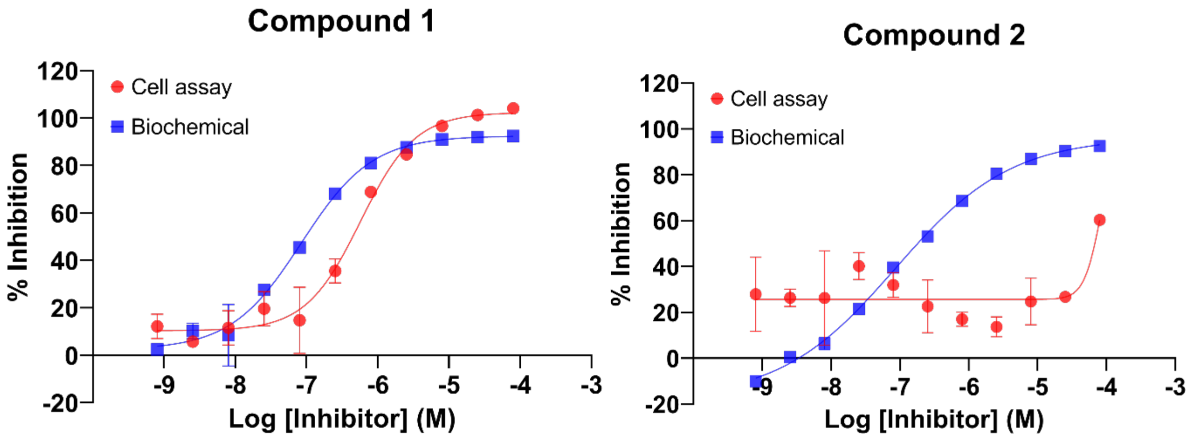

Supplementary Figure 1. Representative HSD17B13 inhibition responses of compound **1** and **2** in biochemical (blue squares) and cell assay (red circles). The means of replicated measurements and associated standard errors were plotted. Compound **1** is active in cell assay (IC_{50} = 0.57 ± 0.12 μM, n=2 independent measurements) but compound **2** is inactive in cell assay (IC_{50}>70 μM, n=4 independent measurement).

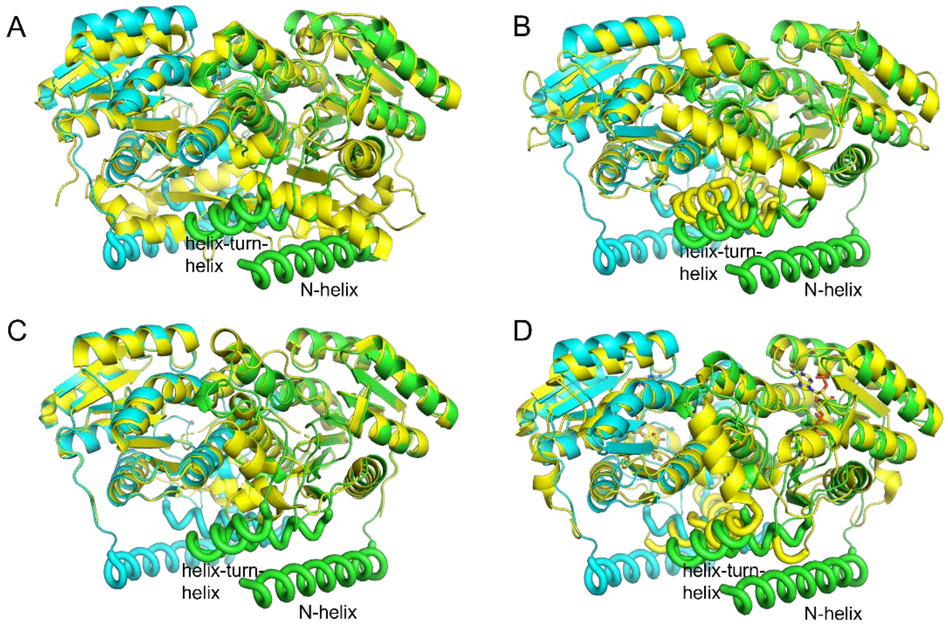

Supplementary Figure 2. The N-terminal membrane anchoring helices are unique in HSD17B13 structure (green and cyan ribbons for HSD17B13 dimer). The N-terminal peptide and the amphipathic helix-turn-helix (tubes) are labeled. Superimposed with (yellow ribbons): A, HSD17B4 (PDB ID 1ZBQ), a cytosolic HSD, which lacks both the N-terminal membrane anchoring helices and the helix-turn-helix motifs; B, HSD17B1 (PDB ID 1QYV), a membrane associated and cytosolic HSD which has a helix-turn-helix but lacks the N-terminal helix; C, HSD17B11 (PDB ID 1YB1), a LD associating HSD but the putative membrane anchoring N-terminal helices and the C-terminal peptide including second helix of the helix-turn-helix motif were truncated in the construct; D, HSD11B1 (PDB ID 1XSE), an ER associating HSD, the N-terminal transmembrane helix was truncated in the construct.

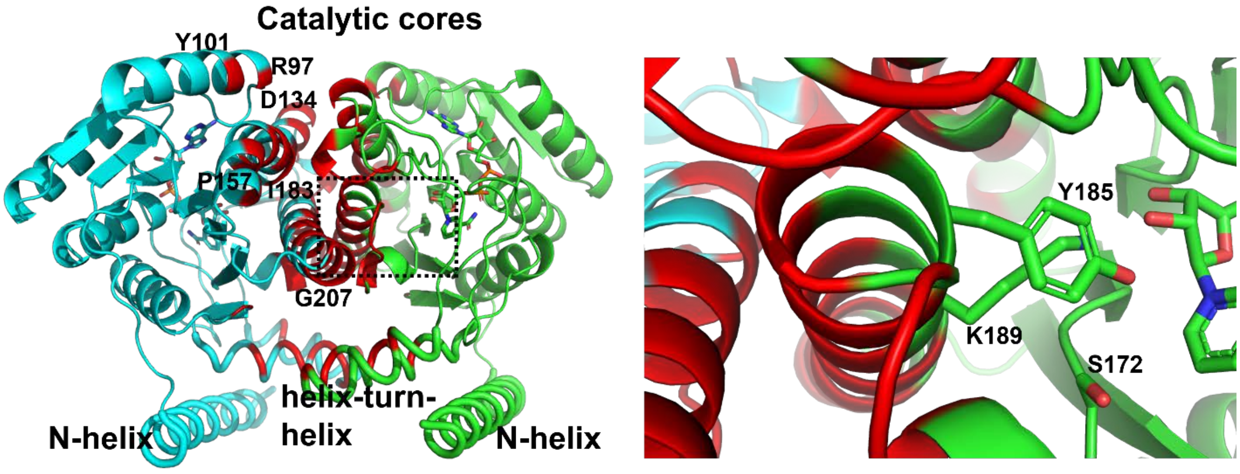

Supplementary Figure 3. HSD17B13 dimer interface. The two subunits of HSD17B13 are shown in green and cyan ribbons, respectively, with residues at the dimer interface in red. The N-terminal helices, the amphipathic helix-turn-helix motifs (tubes), and the catalytic cores are labeled. Residues and peptides of at the dimer interface are labelled for one of the subunits. On the right is the zoom in view of the region in one subunit containing the bound NAD cofactors and the catalytic triad, S172, Y185 and K189 (sticks).

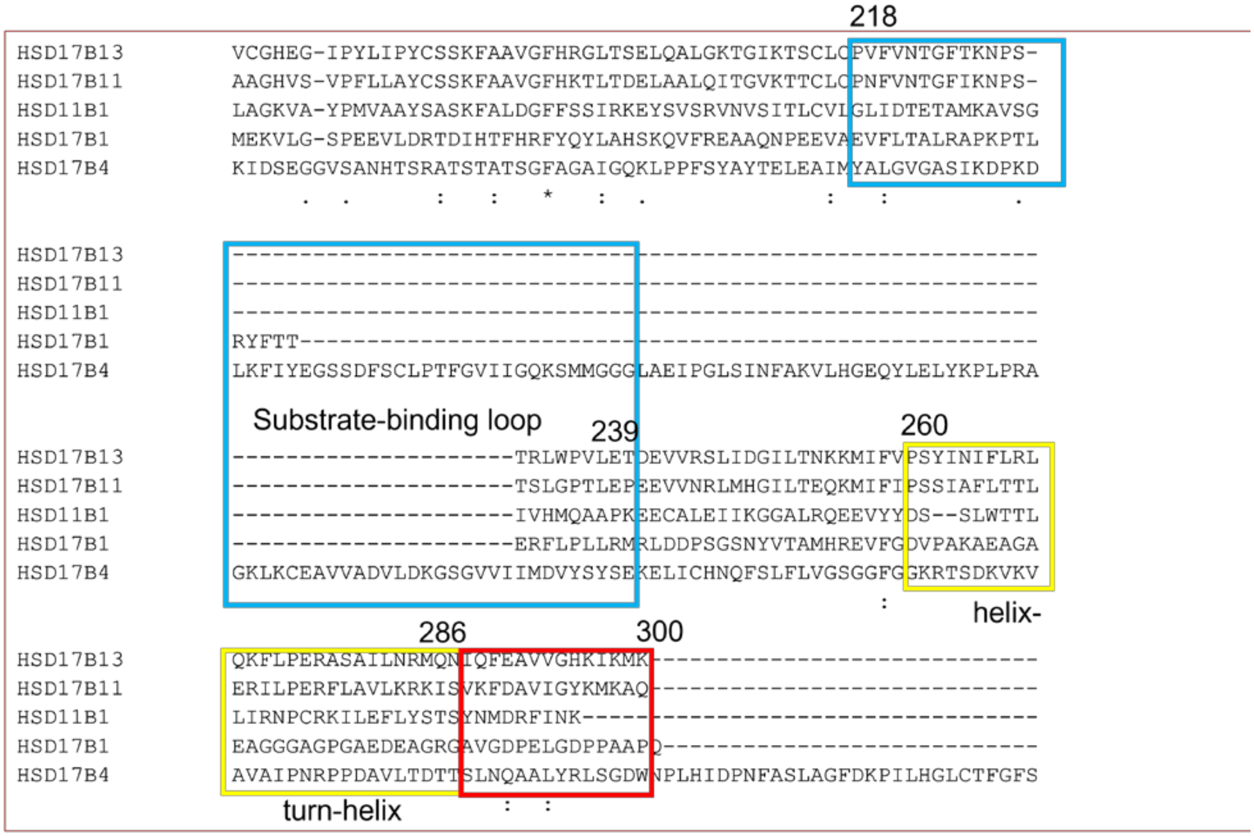

Supplementary Figure 4. Sequence alignments of the C-terminal half of HSD17B13 containing the substrate-binding loop (P218-T239, inside blue box), the helix-turn-helix motif (P260-N286, yellow box), and the C-terminal peptide (red box) with those of representative HSD proteins. The substrates-binding loops and C-terminal peptides of HSD proteins have variable lengths and little sequence conservations HSD17B4 is a much large protein with 711 residues, while other HSDs have 292-328 residues).

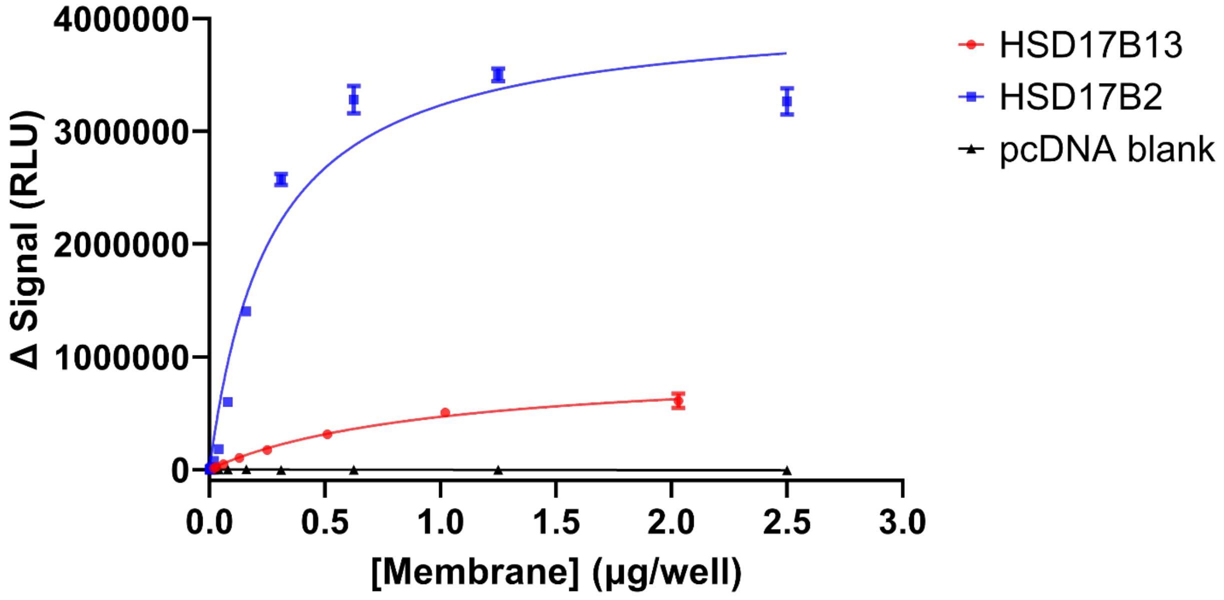

Supplementary Figure 5. Activity comparison between HSD17B13 (red circles) and HSD17B2 (blue squares) measured in NAD(P)H-Glo biochemical assay detecting the luminescent signals of NAD(P)H, using β-estradiol as substrate. Non protein expression cells (pcDNA, black triangles) were used as negative controls. The change in relative luminescent units (RLUs) signals (delta, or Δ) were generated by subtracting background (non- β-estradiol wells) from the wells containing β-estradiol. Representative experiments of replicated measurements (n=2 independent experiments) were shown with mean and standard error bars. The amount of membrane on X-axis is the amount of membrane proteins determined using a Bradford protein assay. The expression level of HSD17B13 and HSD17B2 are comparable judged by protein band intensities on gels.

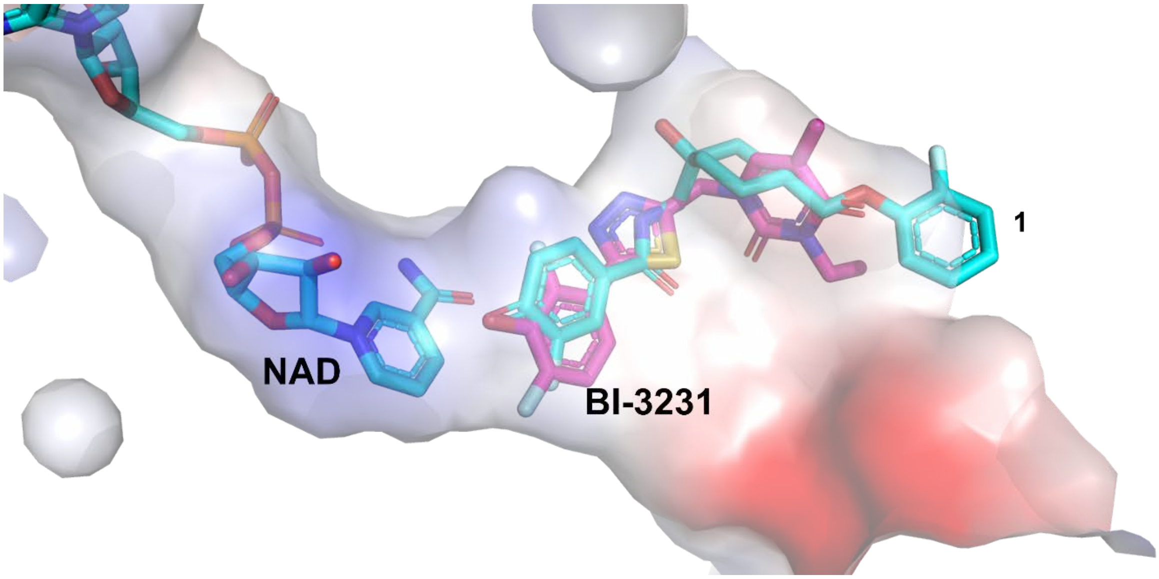

Supplementary Figure 6. Docking of BI-3231 into compound 1 binding site of HSD17B13 (semi-transparent surface). In this docking model, the hydroxyl of BI-3231 overlays with that of compound **1**, and the di-fluorophenol makes similar stacking interaction with the nicotinamide of cofactor NAD^+.

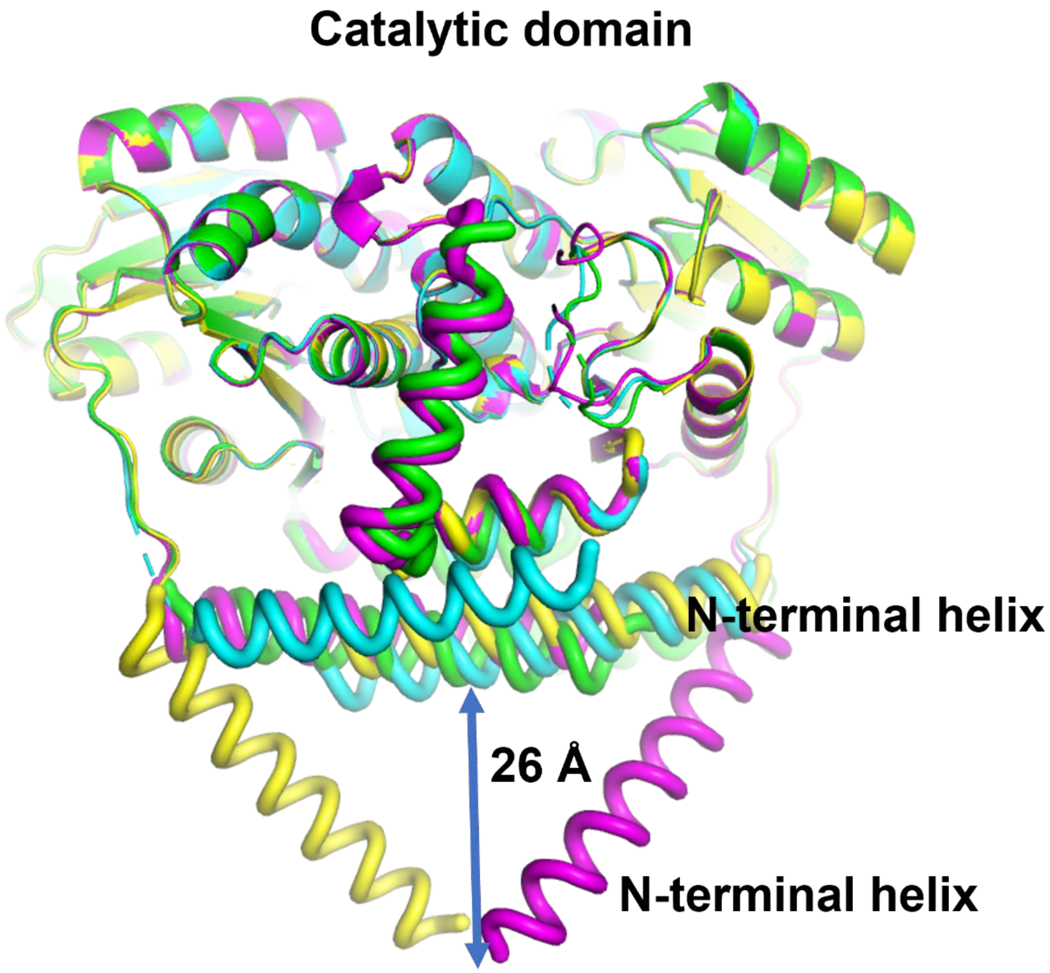

Supplementary Figure 7. Overlay of four independent copies of the human HSD17B13 /compound **2** complex structures in the crystallographic asymmetric unit. The four HSD17B13 dimers are colored green, cyan, magenta and yellow, respectively. The membrane domains (N-terminal helices and the helix-turn-helix motifs) are shown in tubes. The membrane anchoring N-terminal helices and the catalytic cores are labeled. The distance highlighted was measured after transforming the overlapped structures with the N-terminal helices at the lowest position at the Y axis (down in paper), then measured from Y coordinates between the lowest Cα positions of the tilted N-terminal helices and those of the horizontal N-terminal helices.

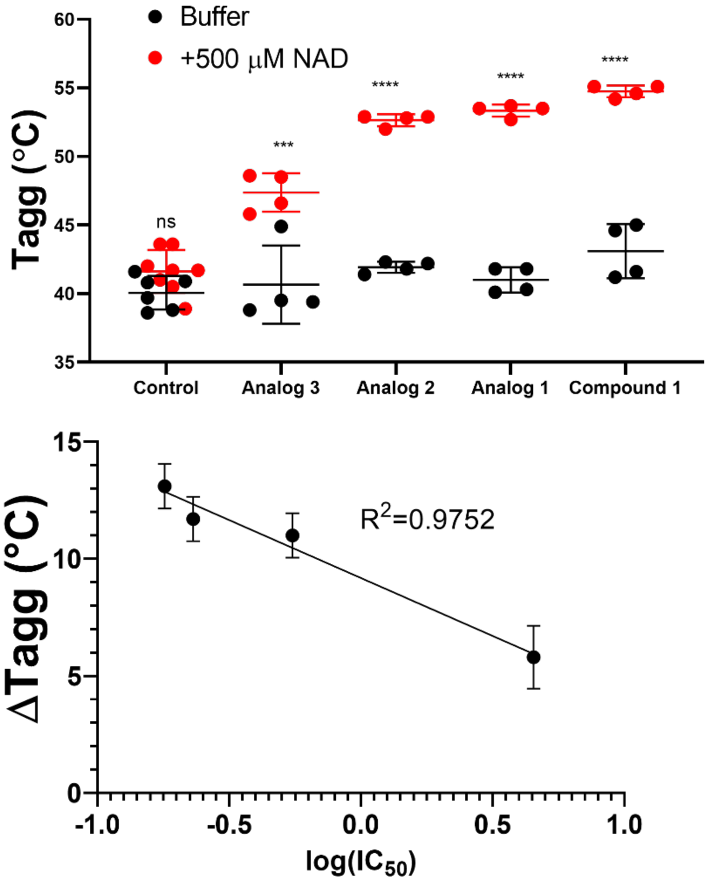

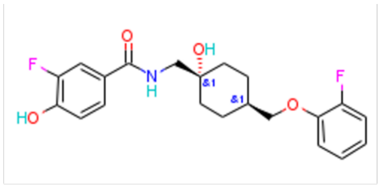

Compound **1** $IC_{50} = 0.18 \pm 0.05$ μM (n=9)

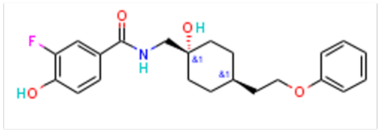

Analog 1 $IC_{50} = 0.23 \pm 0.08$ μM (n=6)

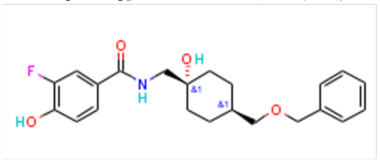

Analog 2 $IC_{50} = 0.55 \pm 0.12$ μM (n=9)

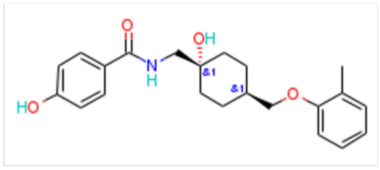

Analog 3 $IC_{50} = 4.5 \pm 0.9$ μM (n=4)

Supplementary Figure 8. The degree of stabilization of human HSD17B13 by compounds measured in thermal shift assay was correlated to their IC_{50} values. Data are presented as mean values ± SEM. 100 μM ligands and 10.4 μM protein were used in the experiments (n≥4 independent measurements for each condition). Control: buffer with (red circles)/without 500 μM NAD^+ (black circles) added. ΔTagg values and two-sided unpaired t test statistics were calculated using Tagg(compound + NAD^+) (red circles)-Tagg(buffer + NAD^+) (black circles). The p values are 0.066, 0.005, <0.0001, <0.0001, <0.001, for control, analogs 3, 2, 1 and compound **1**, respectively. The R^2 of simple linear regression was given for fitting ΔTagg and $\log_{10}(IC_{50})$. Statistic symbols, ns, $p>0.05$; ***, $p=0.005$; ****, $p<0.0001$.

Residues	Variant Type	Expression and Purification Observations
1-288	Truncation	Reasonable yield. Screened for crystallization
5-288	Truncation	Similar to 1-288
8-288	Truncation	Reduced expression
11-288	Truncation	Significantly reduced expression
14-288	Truncation	Significantly reduced expression
17-288	Truncation	No detected expression
21-288	Truncation	No detected expression
20-300	Truncation	Protein expressed but insoluble
1-27 – Protease site - 28-300	Insertion	No detected expression
1-32 - Protease site – 32-300	Insertion	No detected expression
1-271 + VSS (IsoD)	Truncation	Reduced expression, protein yield, and activity
1-300; P260S	Mutation	Reduced expression. Protein insoluble
1-300; Y185F or Y185A	Mutation	Reduced protein solubility and stability
1-300; K189A	Mutation	Reasonable yield but shifted size exclusion profile
1-300; F25E/F26E	Mutation	Reduced expression. Protein insoluble
1-300; F25A/F26A	Mutation	Reasonable yield. Screened for crystallization
1-300; T138R	Mutation	Broad size exclusion profile. Aggregated during concentration
1-300; L281E	Mutation	Low purification yield and poor stability

Supplemental Table S1. Example of some of the human HSD17B13 truncations and variants tested during initial construct screening for crystallization. While some truncation at the N- and C-termini was tolerated, >5AA at the N-terminus appeared to impact expression and abrogated it completely by ~20AA. Different variants were also tested. As has previously been reported, the P260S mutation and IsoD truncation resulted in reduced protein expression and solubility.

Supplementary Method:

General Information: Silica gel flash chromatography was performed with RediSep® Rf Gold normal phase silica flash columns on a CombiFlash® Rf system from Teledyne Isco, Inc. ^{1}H and ^{13}C nuclear magnetic resonance (NMR) spectra were recorded on a Bruker 400 MHz spectrometer. Carbon (^{13}C) NMR spectra are proton decoupled. Chemical shifts are reported in ppm relative to dimethyl sulfoxide (^{1}H NMR δ 2.50; ^{13}C NMR δ 39.52). Multiplicities are denoted as follows: s, singlet; d, doublet; br. s., broad singlet). High-resolution mass spectra (HRMS) were acquired on a Sciex model 5600+ QToF (ToF).

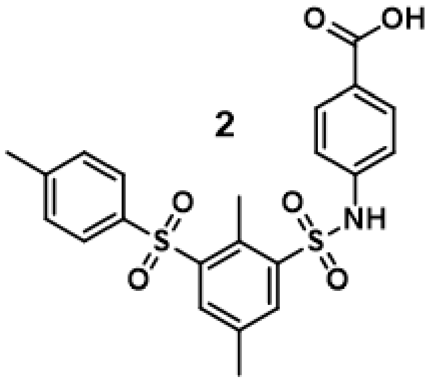

2

4-((2,5-dimethyl-3-tosylphenyl)sulfonamido)benzoic acid (2): Compound **2** (26.5 mg, 57.6 mmol) was obtained from the legacy Pfizer compound collection and was repurified by flash column chromatography (4g silica gel, 0à20% methanol in dichloromethane gradient) to afford **2** (19.1 mg, 41.8 mmol) as a white solid. ^{1}**H NMR** (400 MHz, DMSO-*d*6): δ 12.49 (br. s, 1H), 10.82 (br. s, 1H), 8.19 (d, *J* = 1.9 Hz, 1H), 8.11 (d, *J* = 1.8 Hz, 1H), 7.75 (d, *J* = 8.5 Hz, 2H), 7.64 (d, *J* = 8.0 Hz, 2H), 7.38 (d, *J* = 7.9 Hz, 2H), 7.05 (d, *J* = 8.5 Hz, 2H), 2.58 (s, 3H), 2.46 (s, 3H), 2.40 (s, 3H). ^{13}**C NMR** (DMSO-*d*6, 101 MHz): δ 166.6, 144.6, 141.2, 141.2, 140.0, 137.5, 137.1, 135.2, 133.9, 133.2, 130.8, 130.1, 127.2, 125.5, 117.5, 21.0, 20.4, 15.0. **HRMS:** Calculated for $C_{22}H_{22}NO_6S2$ $[M+H]^+$: 460.0883, found 460.0884.

^{1}H NMR of Compound 2 in DMSO-*d6* (400 MHz)

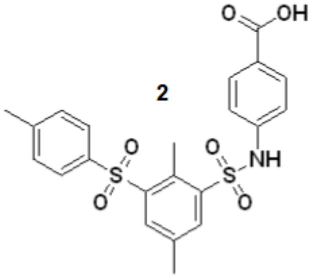

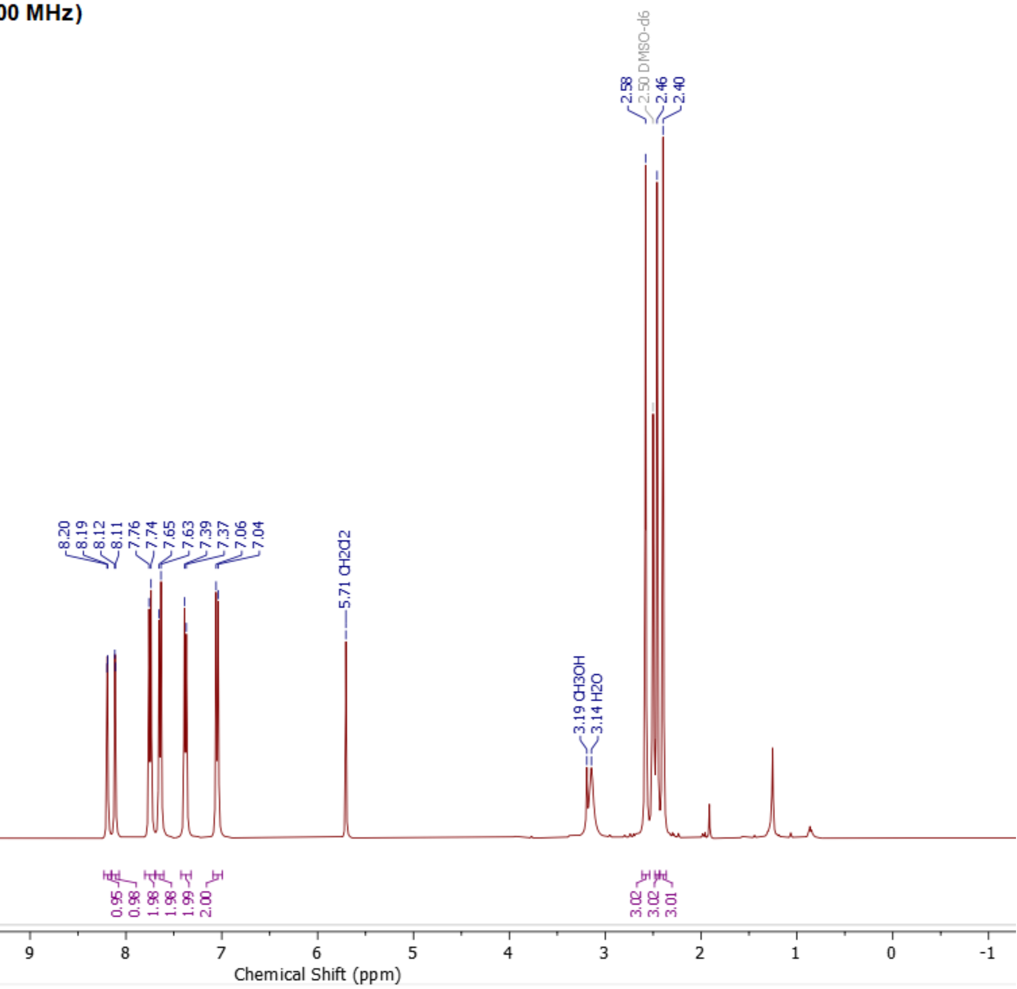

^{13}C NMR of Compound 2 in DMSO-*d6* (101 MHz)

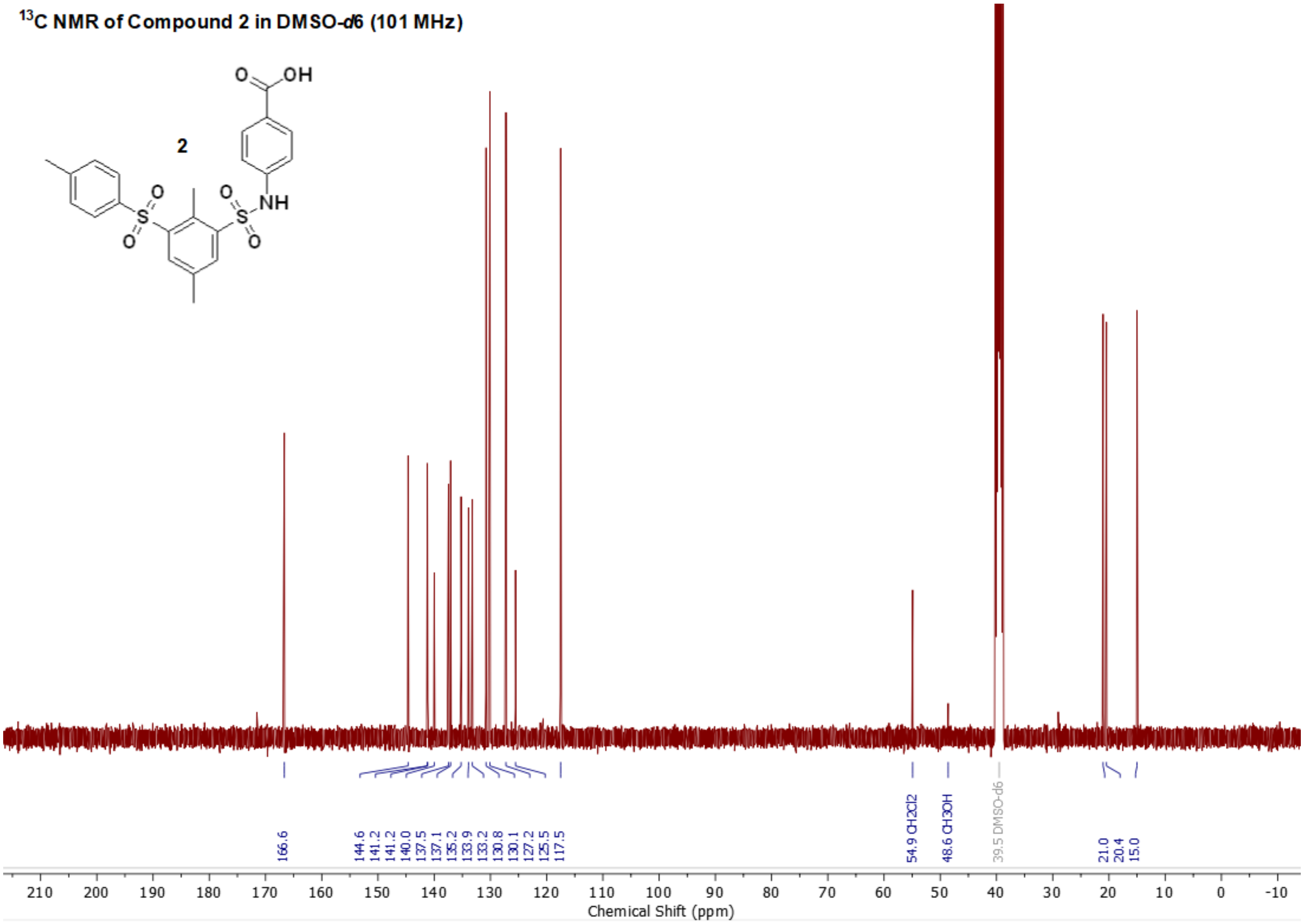

Supplement: Supplementary file 1 — Supplementary Information [file 41467_2023_40766_MOESM1_ESM.pdf]
