# Supplementary material for: Structural basis of lipid-droplet localization of 17-beta-hydroxysteroid dehydrogenase 13
Source: Nat Commun. 2023 Aug 24;14:5158. doi: 10.1038/s41467-023-40766-0 (PMC10449848; doi:10.1038/s41467-023-40766-0)
Supplement: Supplementary file 3 — Reporting Summary [file 41467_2023_40766_MOESM3_ESM.pdf]

nature portfolio

Corresponding author(s): Shenping Liu, Ruth Sommese

Last updated by author(s): 2023/08/01

Reporting Summary

Nature Portfolio wishes to improve the reproducibility of the work that we publish. This form provides structure for consistency and transparency in reporting. For further information on Nature Portfolio policies, see our Editorial Policies and the Editorial Policy Checklist.

Statistics

For all statistical analyses, confirm that the following items are present in the figure legend, table legend, main text, or Methods section.

n/a	Confirmed	
☐	☒	The exact sample size (*n*) for each experimental group/condition, given as a discrete number and unit of measurement
☐	☒	A statement on whether measurements were taken from distinct samples or whether the same sample was measured repeatedly
☐	☒	The statistical test(s) used AND whether they are one- or two-sided<br>*Only common tests should be described solely by name; describe more complex techniques in the Methods section.*
☒	☐	A description of all covariates tested
☒	☐	A description of any assumptions or corrections, such as tests of normality and adjustment for multiple comparisons
☐	☒	A full description of the statistical parameters including central tendency (e.g. means) or other basic estimates (e.g. regression coefficient) AND variation (e.g. standard deviation) or associated estimates of uncertainty (e.g. confidence intervals)
☐	☒	For null hypothesis testing, the test statistic (e.g. *F*, *t*, *r*) with confidence intervals, effect sizes, degrees of freedom and *P* value noted<br>*Give P values as exact values whenever suitable.*
☒	☐	For Bayesian analysis, information on the choice of priors and Markov chain Monte Carlo settings
☒	☐	For hierarchical and complex designs, identification of the appropriate level for tests and full reporting of outcomes
☒	☐	Estimates of effect sizes (e.g. Cohen's *d*, Pearson's *r*), indicating how they were calculated

Our web collection on statistics for biologists contains articles on many of the points above.

Software and code

Policy information about availability of computer code

Data collection	XDS (Built 20220220), autoPROC of Globalphasing version 2023, UNcle software version 4.0 (Unchained Labs, CA, USA) ,Envision plate reader V1.12 (Perkin Elmer)
Data analysis	Phaser of CCP4 7/8, Buster of Globalphasing version 2023, Coot 8.1, Pymol (V2.3.1), GraphPad Prism 9, PISA version 2.1.0

For manuscripts utilizing custom algorithms or software that are central to the research but not yet described in published literature, software must be made available to editors and reviewers. We strongly encourage code deposition in a community repository (e.g. GitHub). See the Nature Portfolio guidelines for submitting code & software for further information.

Data

Policy information about availability of data

All manuscripts must include a data availability statement. This statement should provide the following information, where applicable:

- Accession codes, unique identifiers, or web links for publicly available datasets
- A description of any restrictions on data availability
- For clinical datasets or third party data, please ensure that the statement adheres to our policy

The atomic coordinates and structure factors (PDB ID 8G84 https://doi.org/10.2210/pdb8G84/pdb, 8G89 https://doi.org/10.2210/pdb8G89/pdb, 8G93 https://doi.org/10.2210/pdb8G93/pdb and 8G9V https://doi.org/10.2210/pdb8G9V/pdb) have been deposited in the Protein Data Bank, Research Collaboratory for Structural Bioinformatics, Rutgers University, New Brunswick, NJ (http://www.rcsb.org/).

Human research participants

Policy information about studies involving human research participants and Sex and Gender in Research.

Reporting on sex and gender	The manuscript does not involve human research participants
Population characteristics	N/A
Recruitment	N/A
Ethics oversight	N/A

Note that full information on the approval of the study protocol must also be provided in the manuscript.

Field-specific reporting

Please select the one below that is the best fit for your research. If you are not sure, read the appropriate sections before making your selection.

☒ Life sciences ☐ Behavioural & social sciences ☐ Ecological, evolutionary & environmental sciences

For a reference copy of the document with all sections, see nature.com/documents/nr-reporting-summary-flat.pdf

Life sciences study design

All studies must disclose on these points even when the disclosure is negative.

Sample size	We did not calculate the sample sizes for our experiments. Each experiment was based on the average of replicated measurements, and the each experiment was replicated at least 3 rimes. The consistency between experiments determine the sample sizes we use.
Data exclusions	No data were excluded from analysis.
Replication	Replication of experiments were successful
Randomization	We had positive and negative controls in our experiments. Our experiments are in vitro experiments and randomization is not needed.
Blinding	Our experiments are all in vitro experiments and no need for blinding.

Reporting for specific materials, systems and methods

We require information from authors about some types of materials, experimental systems and methods used in many studies. Here, indicate whether each material, system or method listed is relevant to your study. If you are not sure if a list item applies to your research, read the appropriate section before selecting a response.

Materials & experimental systems

n/a	Involved in the study	
☒	☐	Antibodies
☐	☒	Eukaryotic cell lines
☒	☐	Palaeontology and archaeology
☒	☐	Animals and other organisms
☒	☐	Clinical data
☒	☐	Dual use research of concern

Methods

n/a	Involved in the study	
☒	☐	ChIP-seq
☒	☐	Flow cytometry
☒	☐	MRI-based neuroimaging

Eukaryotic cell lines

Policy information about cell lines and Sex and Gender in Research

Cell line source(s)	HEK293 and Spodoptera frugiperda cells for protein expression
Authentication	HEK293 cells are commercial available for routine protein expression. No authentication is needed.
Mycoplasma contamination	cells were not tested for mycoplasma contamination

Commonly misidentified lines
(See ICLAC register)

none
